# Deep learning-based recognition of key anatomical structures during robot-assisted minimally invasive esophagectomy

**DOI:** 10.1007/s00464-023-09990-z

**Published:** 2023-03-22

**Authors:** R. B. den Boer, T. J. M. Jaspers, C. de Jongh, J. P. W. Pluim, F. van der Sommen, T. Boers, R. van Hillegersberg, M. A. J. M. Van Eijnatten, J. P. Ruurda

**Affiliations:** 1 Department of Surgery, University Medical Center Utrecht, Heidelberglaan 100, 3584 CX Utrecht, The Netherlands; 2 Department of Biomedical Engineering, Eindhoven University of Technology, Groene Loper 3, 5612 AE Eindhoven, The Netherlands; 3 Department of Electrical Engineering, Eindhoven University of Technology, Groene Loper 19, 5612 AP Eindhoven, The Netherlands

**Keywords:** Surgery, Anatomy recognition, Deep learning, Computer vision, Robotics

## Abstract

**Objective:**

To develop a deep learning algorithm for anatomy recognition in thoracoscopic video frames from robot-assisted minimally invasive esophagectomy (RAMIE) procedures using deep learning.

**Background:**

RAMIE is a complex operation with substantial perioperative morbidity and a considerable learning curve. Automatic anatomy recognition may improve surgical orientation and recognition of anatomical structures and might contribute to reducing morbidity or learning curves. Studies regarding anatomy recognition in complex surgical procedures are currently lacking.

**Methods:**

Eighty-three videos of consecutive RAMIE procedures between 2018 and 2022 were retrospectively collected at University Medical Center Utrecht. A surgical PhD candidate and an expert surgeon annotated the azygos vein and vena cava, aorta, and right lung on 1050 thoracoscopic frames. 850 frames were used for training of a convolutional neural network (CNN) to segment the anatomical structures. The remaining 200 frames of the dataset were used for testing the CNN. The Dice and 95% Hausdorff distance (95HD) were calculated to assess algorithm accuracy.

**Results:**

The median Dice of the algorithm was 0.79 (IQR = 0.20) for segmentation of the azygos vein and/or vena cava. A median Dice coefficient of 0.74 (IQR = 0.86) and 0.89 (IQR = 0.30) were obtained for segmentation of the aorta and lung, respectively. Inference time was 0.026 s (39 Hz). The prediction of the deep learning algorithm was compared with the expert surgeon annotations, showing an accuracy measured in median Dice of 0.70 (IQR = 0.19), 0.88 (IQR = 0.07), and 0.90 (0.10) for the vena cava and/or azygos vein, aorta, and lung, respectively.

**Conclusion:**

This study shows that deep learning-based semantic segmentation has potential for anatomy recognition in RAMIE video frames. The inference time of the algorithm facilitated real-time anatomy recognition. Clinical applicability should be assessed in prospective clinical studies.

**Supplementary Information:**

The online version contains supplementary material available at 10.1007/s00464-023-09990-z.

Esophageal cancer is the 8th most common cancer worldwide and curative treatment consists of neoadjuvant chemoradiatherapy followed by surgical resection [1, 2]. Minimally invasive esophagectomy, either robot-assisted or via a conventional thoracolaparoscopic approach, has been increasingly adopted as the preferred approach for surgical resection of esophageal cancer in recent years [3–6]. Robot-assisted surgery allows a wide range of motion, a stable, magnified and three-dimensional optical system, and tremor suppression. However, as illustrated by its learning curve of 24–70 cases, robot-assisted minimally invasive esophagectomy (RAMIE) is a highly complex procedure [7–9].

Especially for novice surgeons, recognition of key anatomical structures during RAMIE remains challenging. Although the zoomed in operating view is valuable for detailed vision and accurate surgical dissection, it poses challenges for surgical orientation and maintaining an overview over the operating field. Additional assistance in anatomical and surgical orientation is therefore warranted. RAMIE is performed through a different anatomical view than open esophagectomy. Assisting surgeons during the transition from open to minimally invasive surgery by recognizing anatomical landmarks could help in avoiding damage to vital structures and may thereby improve perioperative surgical outcomes, which could result in a reduction of the RAMIE learning curve. Intraoperative complications are still an unsolved problem and an estimated 20% of adverse events are caused by misrecognition [10].

Deep learning has substantially advanced the state-of-the art in numerous medical imaging problems [11–13]. However, a recent systematic review showed that deep learning-based anatomy recognition on surgical videos is a research field that is still in its infancy [14]. Previous studies have mostly focused on recognition of anatomical structures in laparoscopic cholecystectomy, a commonly performed procedure of lesser complexity. No studies are published regarding recognition of key anatomical structures in complex oncological thoracic surgery, such as RAMIE. Computer-aided anatomy recognition may be particularly useful for complex robot-assisted surgery, given the substantial learning curves and complex surgical orientation. This is essential due to the vital anatomical structures situated within the narrow operating field, including the aorta, trachea, and the azygos vein. Computer-aided anatomy recognition is facilitated through the surgical robot which has an interface to apply this technology.

This study’s objective was to develop a real-time anatomy recognition algorithm for thoracoscopic video frames from RAMIE procedures using deep learning. Specifically, a deep convolutional neural network (CNN) was trained to segment the azygos vein and/or vena cava, aorta, and right lung on the intraoperative frames.

##  Methods

###  Study design

A retrospective single-center cohort study was conducted at University Medical Center (UMC) Utrecht, The Netherlands. Surgical videos were collected from consecutive patients between January 2018 and July 2021 who underwent a RAMIE procedure for esophageal cancer, with or without neoadjuvant chemoradiotherapy according to the CROSS-regimen [15]. Patients without a surgical video of the thoracic phase were excluded. Ethical approval was provided by the Institutional Review Board of UMC Utrecht (Approval Number 22/634) and informed consent was waived. The procedures were performed by two expert RAMIE surgeons (more than 200 RAMIE procedures each). Videos were recorded with a frame rate of 25 Hz and a resolution of 960 × 540 pixels. To enable efficient further processing by the convolutional neural network (CNN), all frames were rescaled to a standard resolution of 256 × 256 pixels, and black edges around the frames were removed.

### Annotations

A total of 1050 frames were labeled by a PhD candidate in esophageal surgery (RdB). This included two feedback sessions with revision of complex as well as random frames by an expert upper gastrointestinal surgeon (JR). Frames were manually selected with the criteria that either the azygos vein, vena cava, aorta, and/or lung were visible. Lymphatic or fatty tissue was excluded in the annotation of the anatomy. The vena cava and azygos vein were considered one class and were visible on 1035 frames, whereas the aorta was located on 343 frames and the lung on 397 frames. Variation of complexity of the anatomical situation was included in the selected frames to improve algorithm performance for a broad anatomical variety. All anatomical structures on the frames were labeled using the LabelMe framework [16]. To assess the variation in the labeled dataset, a small randomly chosen subset consisting of 25 frames were annotated twice in random order with seven days in between. The subset contained 23 frames where the vena cava and azygos vein were visible, four frames included the aorta, and 15 showed the lung. Additionally, this subset was annotated by the expert surgeon to assess the variability between the novice and expert annotators (RdB and JR). The intra- and interobserver variation are expressed in terms of Dice scores and 95% Hausdorff distances, further explained in the subsection “outcome measures.”

### CNN architecture and training

**Fig. 1:**
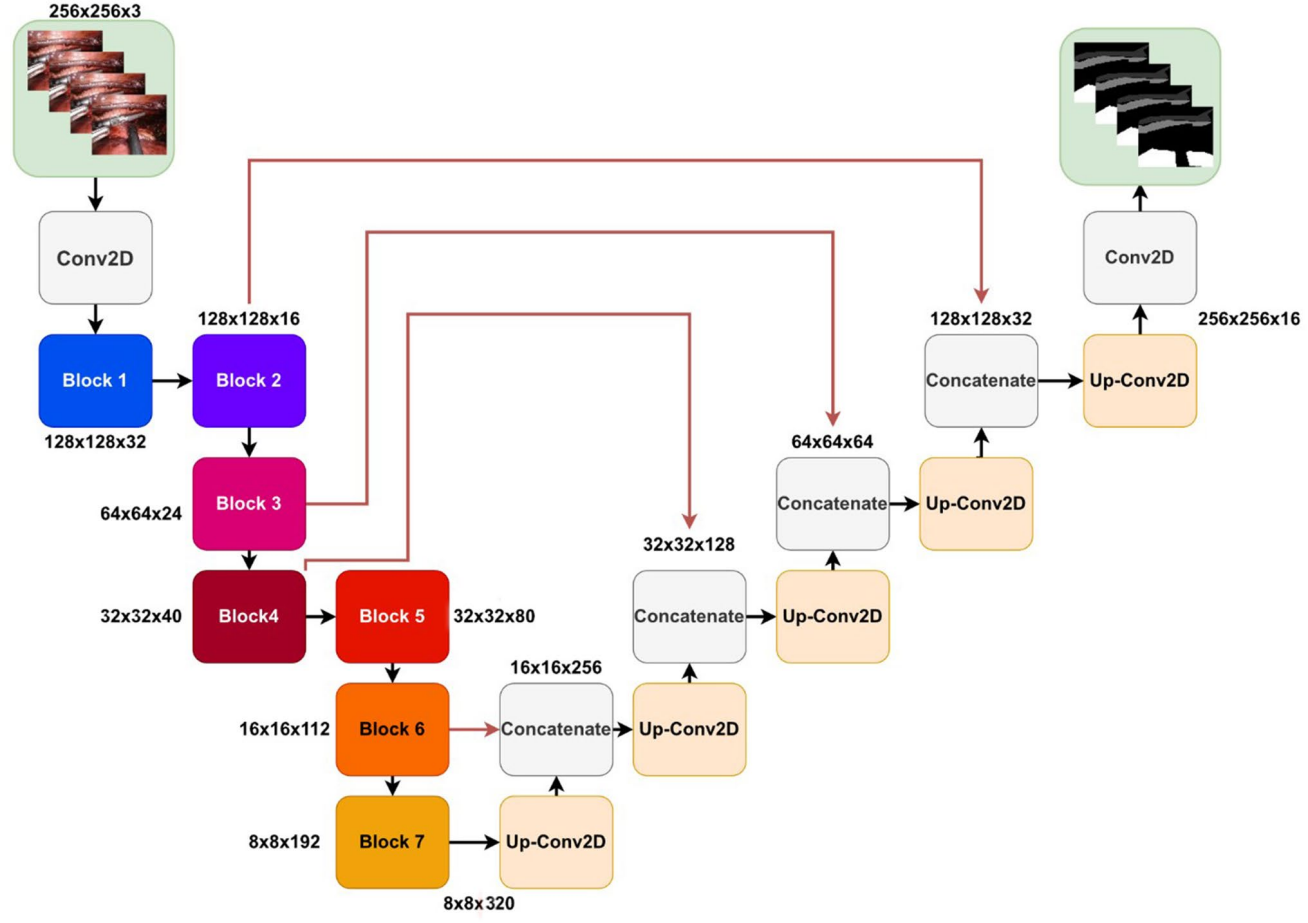
A schematic representation of the U-net-like model with an EfficientNet-b0 encoder. Each block corresponds to a multi-channel feature map. The number of channels is given at each block. The exact composition of all blocks can be found in the supplementary material

To extract anatomical information from the intraoperative frames, a CNN was trained to segment the azygos vein and/or vena cava, aorta, and lung. The labeled dataset ‘**A**’ was randomly split on patient level into a set for model training and testing. The training set contained 850 frames from 66 patients (80%), which was further subdivided into five folds for cross-validation. The vena cava and/or azygos vein were annotated on 838 (99%) frames, the aorta on 279 (33%) frames, and the lung on 302 (36%) frames. Figure 1 shows the proposed network architecture. The architecture was based on the original U-net, since it has proven to show state-of-the-art performance in numerous medical image segmentation problems [17–19]. EfficientNet-B0 was used as the encoder (details on the architecture can be found in Appendix A), with a total number of 5.84 million trainable parameters [20]. EfficientNet has shown to produce better performance with fewer parameters, decreasing inference time, and shifting model predictions toward real time. We built upon networks implemented in the Pytorch framework [20].

Binary cross-entropy was used as loss function; the definition can be found in Appendix B. The loss was updated for a maximum of 1000 epochs using the Adam optimizer (*β*1 = 0.9, *β*2 = 0.999) with a base learning rate of 0.001 [21]. If no improvement of the validation loss was observed for 10 consecutive epochs, the learning rate was halved and 50 consecutive epochs without improvement was set as an early stopping criterion. The parameters of the epoch where the validation loss last improved were saved.

Data augmentations, commonly used in deep learning applications to increase the amount of input data [22], that were applied to the training frames included translation, rotations, scaling, horizontal, and vertical flipping. Additionally, random blurring, noise, brightness, contrast, and saturation augmentations were applied to improve robustness against differences in recording hardware or lighting. The network was trained using a batch size of 32 frames on a NVIDIA GTX Titan GPU with 12 GB of internal memory.

### Experiments

All experiments were performed on the test set, which included 200 frames from 17 patients (20%), with the vena cava and azygos vein visible on 197 (99%) frames, the aorta on 64 frames (32%), and the lung on 95 (48%) frames. Pretraining and finetuning were used with the goal of reaching higher accuracy without including additional annotated data. The encoder was pretrained on two datasets: ImageNet and GastroNet [23, 24]. ImageNet is a large-scale dataset including more than 1.2 million labeled images and 1000 classes. GastroNet includes more than 5 million unlabeled images and 3675 labeled images categorized in 5 classes. Pretraining on GastroNet was done using a semi-supervised learning method proposed by Xie et al. [25]. Details on the finetuning of the network can be found in Appendix C. These results were compared to a newly initialized network (trained from scratch).

Additionally, the impact of the size of the training data was evaluated as a secondary outcome. All models were also trained on 10% to 100% of the training data in ten steps. Per step five models were trained on a different fraction from the training data. The results were evaluated on Dice and 95% Hausdorff distance.

###  Outcome measures

**Fig. 2:**
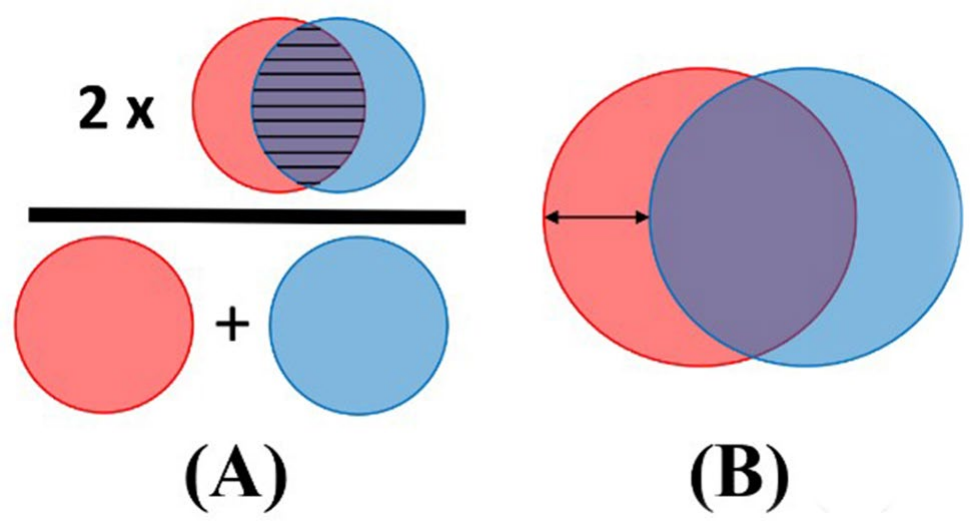
Visual example of the primary evaluation metrics. Subfigure** a** shows how to interpret the Dice and subfigure** b** shows the Hausdorff distance

Primary outcomes were Dice and Hausdorff distances, and secondary outcomes were pixel-wise accuracy, sensitivity, and specificity. The Dice is a metric that represents the total overlap of the prediction by the model and the reference annotation (annotation by the expert RAMIE surgeon) and is calculated as follows:

$$\text{Dice coefficient }=\frac{2|A\cap B|}{|A|+|B|},$$

where *A* and *B* represent two segmentation areas. In case of this study, *A* and *B* represent the predicted segmentation by the deep learning algorithm and the manual reference annotation. A Dice of 1 indicates perfect overlap and 0 indicates no overlap at all. The Hausdorff distance is a measure to indicate the largest distance between a point of the prediction and the reference annotation. In this study the 95% Hausdorff distance (95HD) is calculated as follows:

$$95\mathrm{HD}=\left(\overrightarrow{{d}_{H,95}}(A,B)+\overrightarrow{{d}_{H,95}}(B,A)\right)/2,$$

where *A* and *B* represent the boundaries of the segmented regions. Figure 2 shows a graphical explanation of these evaluation metrics.

### Statistical analysis

A statistical comparison between both pretraining methods and training from scratch was performed using the non-parametric Wilcoxon signed-rank test. A two-sided test was adopted where a *p*-value < 0.05 was considered statistically significant.

## Results

### Patient characteristics

Surgical videos of 83 patients were used to train, test, and validate the algorithm. Patient characteristics are displayed in Table 1. The median age of the patients was 68 years (IQR 13 years) and median BMI was 24.7 (IQR 4.0). Most patients were diagnosed with esophageal adenocarcinoma (66%) and treated with neoadjuvant chemoradiotherapy (*N* = 68, 82%). All patients underwent RAMIE with a transthoracic approach.

**Table 1:** Patient characteristics

Variable	Number of patients (*n* = 83)
Age, median (IQR)	68 (13)
Gender	
Male	61 (74%)
Female	22 (26%)
BMI, median (IQR)	24.7 (4.0)
Histology	
Adenocarcinoma	55 (66%)
Squamous cell carcinoma	24 (29%)
Other	4 (5%)
Tumor location	
Upper esophagus	5 (6%)
Middle esophagus	15 (18%)
Distal esophagus	52 (63%)
Gastroesophageal junction	7 (8%)
Cardia	3 (4%)
cT stage	
T1	5 (6%)
T2	12 (15%)
T3	55 (66%)
T4a	5 (6%)
T4b	6 (7%)
cN stage	
N0	41 (49%)
N1	28 (34%)
N2	9 (11%)
N3	5 (6%)
Neoadjuvant therapy	
Chemoradiotherapy	71 (86%)
Chemotherapy	1 (1%)
None	11 (13%)

*BMI* body mass index, * IQR* interquartile range

###  Evaluation

As shown in Table 2, the pretrained weights achieved better accuracy compared to training from scratch. For the vena cava and/or azygos vein, the highest accuracy was reached using pretrained weights from ImageNet (median Dice of 0.79 (IQR = 0.20), 95HD of 5.22 (IQR = 2.60). In addition, for the aorta and the lung the best accuracy was achieved by ImageNet pretrained weights as well. For the aorta a median Dice 0.74 (IQR = 0.86) and median 95HD of 5.87 (IQR = 3.27) were achieved. For the lung a median Dice of 0.89 (IQR = 0.30) and median 95HD of 5.87 (IQR = 3.27) were obtained with ImageNet pretrained weights.

**Table 2:** Accuracy of the proposed deep learning algorithm on all three structures of interest, including statistical analysis with the Wilcoxon Signed-Rank test of all experiments. Results are given as median (IQR), with the best results shown in bold

Anatomical structures	Weight initialization	Primary outcomes		Pixel-wise		
		Dice	95HD[pixels]	Accuracy	Sensitivity	Specificity
Vena cava\ Azygos vein	Scratch	0.67 (0.28)	6.25 (2.51)	0.95 (0.04)	0.81 (0.26)	0.97 (0.04)
	ImageNet	0.79 (0.20)*	5.22 (2.60)*	0.97 (0.03)*	0.86 (0.18)*	0.98 (0.03)*
	GastroNet	0.75 (0.22)*	5.64 (2.38)*	0.96 (0.03)	0.83 (0.21)*	0.98 (0.03)*
Aorta	Scratch	0.26 (0.54)	8.12 (3.80)	0.94 (0.05)	0.51 (0.91)	0.96 (0.05)
	ImageNet	0.74 (0.86)*	5.87 (3.27)*	0.97 (0.03)*	0.85 (0.81)*	0.98 (0.03)*
	GastroNet	0.67 (0.67)*	5.70 (2.91)*	0.97 (0.03)*	0.84 (0.50)*	0.98 (0.03)*
Lung	Scratch	0.59 (0.76)	7.83 (4.28)	0.93 (0.07)	0.96 (0.32)	0.93 (0.08)
	ImageNet	0.89 (0.30)*	5.57 (4.05)*	0.97 (0.05)*	0.95 (0.07)*	0.98 (0.06)*
	GastroNet	0.86 (0.29)*	5.69 (3.05)*	0.96 (0.04)*	0.94 (0.10)*	0.97 (0.05)*

*BMI* body mass index, *IQR* interquartile range

*P < 0.05

**Fig. 3:**
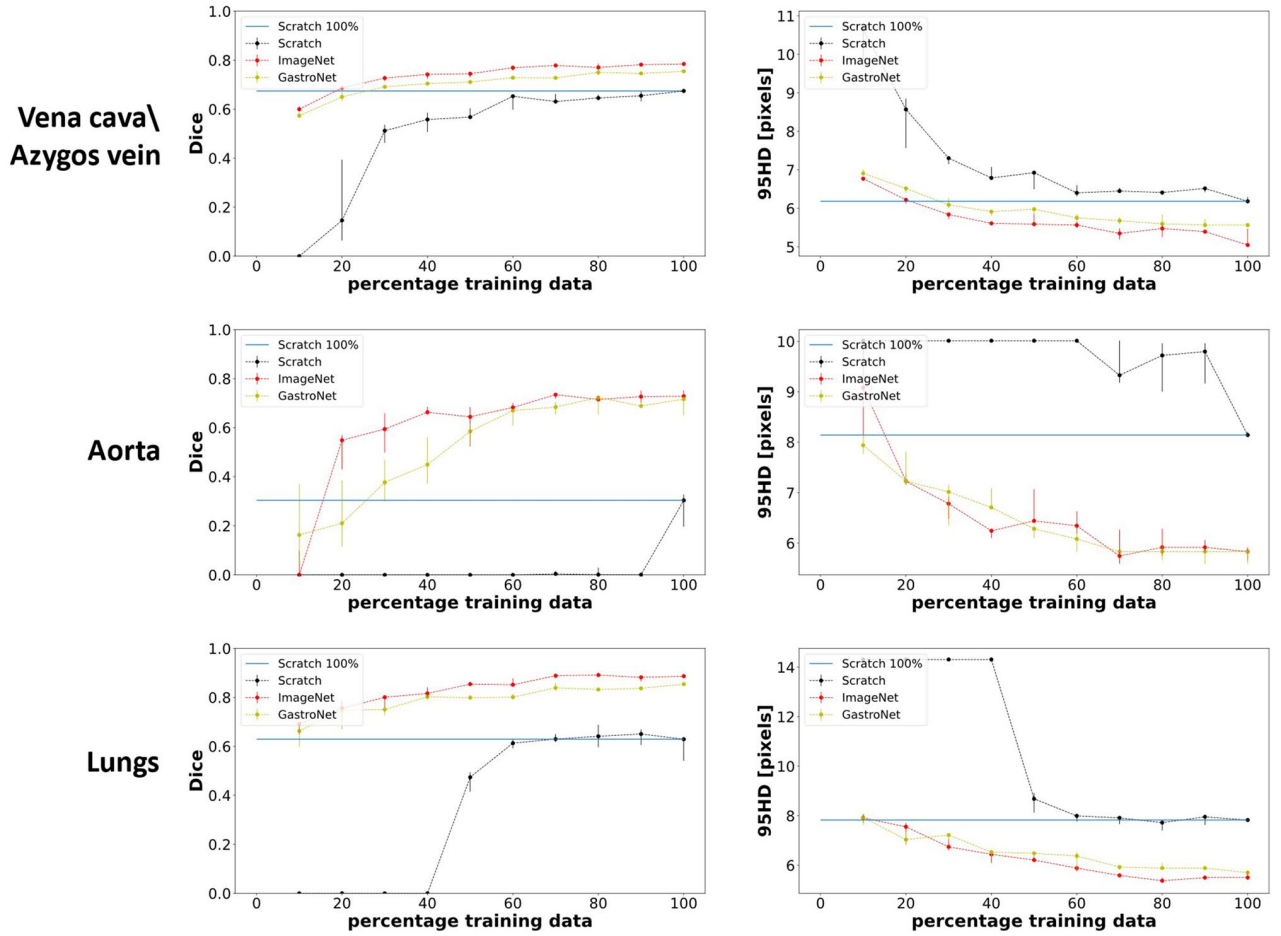
Effect of training size on algorithm accuracy without (black) and with pretraining using ImageNet (red) and GastroNet (yellow) weights. Fivefold cross-validation is used to train the models. The median and the IQR of the median performance of the five models are visualized in the figures. The blue horizontal line indicates model performance trained from scratch on 100% of the training (Color figure online)

The difference between the accuracy of the models with and without pretrained weights increased with a decrease in labeled data (Fig. 3). The blue line in Fig. 3 indicates the highest accuracy of training from scratch on the complete dataset. Regarding the vena cava and azygos vein, the same accuracy could already be achieved with ImageNet pretrained weights using only 20% of the training size (255 frames). Without pretrained weights, the model could not identify the lung using less than 50% of the training data (340 frames). These results are in contrast with the model with pretrained weights from ImageNet and GastroNet, which resulted in a Dice above 0.6 using 10% of the training data. Regarding the aorta, the model trained from scratch needed more than 100% of the training data to get a Dice higher than zero, whereas only 20% of the training data was necessary when applying ImageNet and GastroNet pretrained weights. Additionally training from scratch showed higher variation between model performances trained on a different fraction of the training data. Training with 100% of the data results in almost no variation between the models with pretrained weights, while this is the case for training the model from scratch.

Table 3 shows the intra- and interobserver variation between the surgical PhD candidate, the expert surgeon, and the deep learning algorithm on the randomly selected subset consisting of 25 frames. The highest intraobserver variation in the surgical PhD candidate measured in Dice was found for the vena cava and azygos vein (Dice = 0.89 (IQR = 0.07)). The variation in Dice for the aorta and lung was 0.94 (IQR = 0.01) and 0.97 (IQR = 0.03), respectively. The highest intraobserver variation measured in Hausdorff distance was found for the vena cava and azygos vein (95HD = 3.46 (IQR = 2.68)). In the case of the lung and aorta, the 95HD was 3.10 (IQR = 1.07) and 2.49 (IQR = 1.86). In this same subset, the accuracy of the deep learning algorithm and the surgical PhD candidate was also evaluated in comparison to labeling by an expert surgeon. The median Dice between the expert surgeon and the PhD candidate were 0.86 (IQR = 0.11), 0.94 (IQR = 0.03), and 0.97 (IQR = 0.02) for the vena cava or azygos vein, aorta, and lung, respectively. The prediction of the deep learning algorithm was compared with the expert surgeon annotations, showing an accuracy measured in median Dice of 0.70 (IQR = 0.19), 0.88 (IQR = 0.07), and 0.90 (0.10) for the vena cava or azygos vein, aorta, and lung, respectively.

**Table 3:** Intraobserver variation and interobserver variation between surgical PhD candidate and expert surgeon indicated in Dice and Hausdorff distance (median (IQR))

Anatomical structures	Intraobserver variation		Interobserver variation			
	Dice PhD-PhD	95HD PhD-PhD	Dice Epert-PhD	Dice Expert-Deep learning	95HD Expert-PhD	95HD Expert-Deep learning
Vena cava\azygos vein	0.89 (0.07)	3.46 (2.68)	0.86 (0.11)	0.70 (0.19)	4.43 (1.87)	5.48 (3.02)
Aorta	0.94 (0.01)	3.10 (1.07)	0.94 (0.03)	0.88 (0.07)	2.94 (1.80)	4.39 (1.91)
Lung	0.97 (0.03)	2.49 (1.86)	0.97 (0.02)	0.90 (0.10)	3.64 (1.82)	4.82 (3.71)

Additionally, the accuracy of the deep learning algorithm was compared with the annotations of the expert surgeon

### Visual representation of the accuracy of the deep learning algorithm

**Fig. 4:**
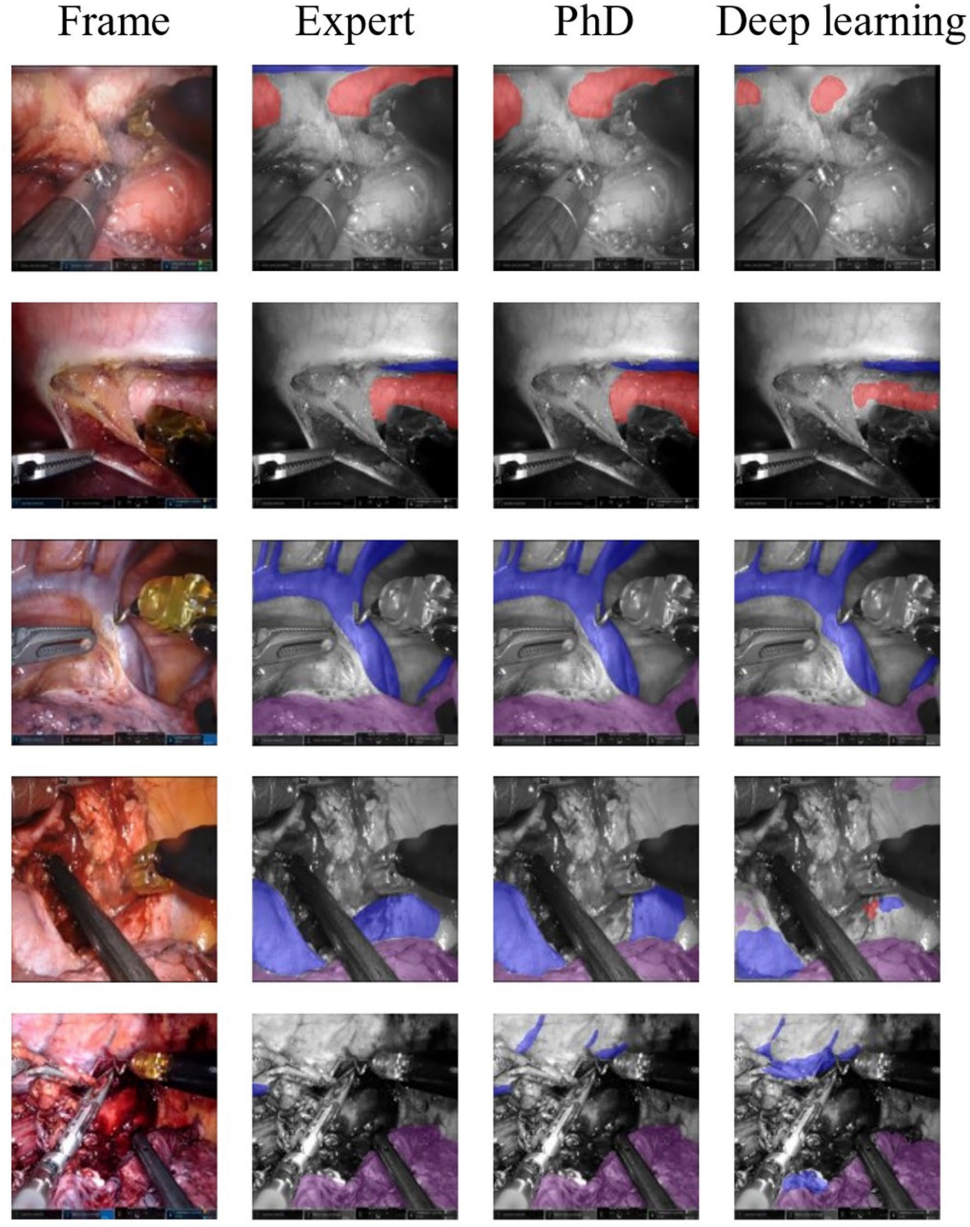
Visual representation of segmentations on randomly selected frames from the test set annotated by an expert (more than 200 RAMIE procedures), PhD (surgical PhD candidate), and the predictions provided by the deep learning algorithm. Vena azygos or vena cava is indicated in blue, aorta in red, and the lung in purple (Color figure online)

**Fig. 5:**
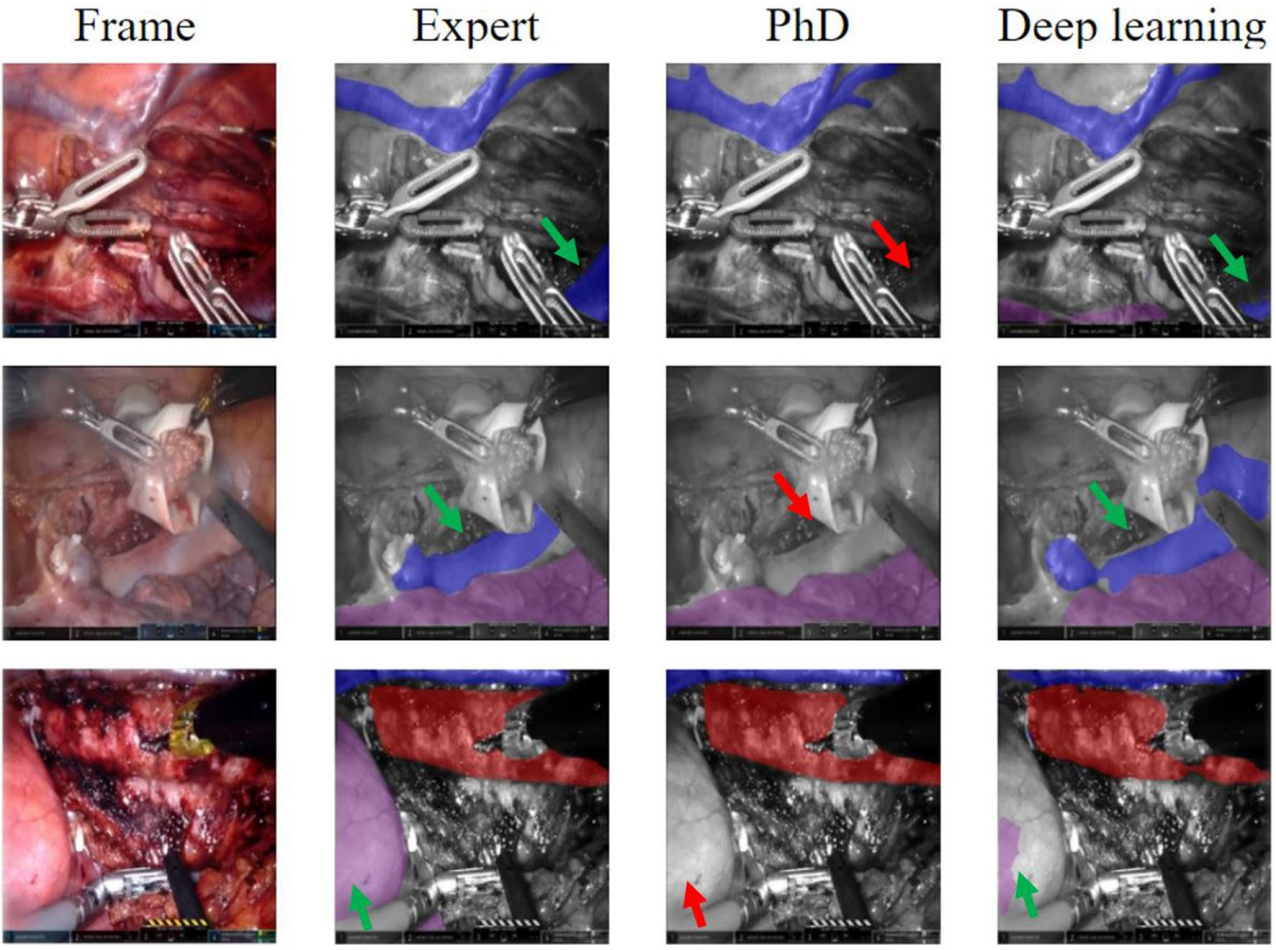
Visual representation of selection of frames with missed structure annotations by the surgical PhD candidate. The annotations by the expert surgeon and the prediction of the deep learning algorithm are displayed for comparison. The vena azygos, vena cava, or lung was missed by the surgical PhD candidate (red arrow) and detected by the expert and the deep learning algorithm (green arrow). The vena azygos or vena cava is indicated in blue, aorta in red, and the lung in purple (Color figure online)

Figure 4 shows five randomly selected example frames from the subset. The annotations from the expert surgeon and the PhD candidate were compared with the predictions of the deep learning algorithm. In the first and third frame, some uncertainties around the edges of the aorta can be detected. Furthermore, on the fifth frame, the deep learning algorithm predicts the appearance of the aorta in the middle of the frames incorrectly. To illustrate the clinical value of our deep learning algorithm, we included frames showing misinterpretation by the surgical PhD candidate and the deep learning algorithm detecting the anatomical structure (Fig. 5). It is observed that the deep learning algorithm detects the azygos vein, vena cava, or lung in the three selected frames, when the surgical PhD candidate did not recognize the structure.

The model processes single frame inputs with an inference speed of 39 frames per second. This shows real-time segmentation using a deep neural network is possible since the surgical videos are recorded with a frame rate of 25 Hz. We included a video of the performance of our deep learning algorithm in the supplementary material.

## Discussion

This study demonstrated that a deep learning-based algorithm can segment key anatomical structures in thoracoscopic RAMIE video frames. The algorithm segments the frames in real time without visible latency with an inference time of 39 frames per second. Our algorithm achieved a median Dice of 0.79 (IQR = 0.20) and a median 95HD of 5.22 (IQR = 2.60) pixels on the test set for azygos vein and vena cava and for segmentation of the aorta a Dice of 0.74 (IQR = 0.86) and 95HD of 5.87 (IQR = 3.27). Segmentation of the lung reached a Dice of 0.89 (IQR = 0.30) and 95HD of 5.57 (IQR = 4.05). Our study is the first to report on deep learning-based segmentation of key anatomical structures in a complex thoracic oncological surgical procedure, such as RAMIE. Furthermore, the current study elaborated on the added value of pretraining for deep learning-based anatomy recognition. Additionally, this study shows that annotation by a surgical PhD candidate under supervision by an expert surgeon is accurate.

Visual examination of segmentations of the vena cava and azygos vein showed that most challenging regions were around the edges and smaller veins are missed in some cases. Uncertainty around the edges could be caused by the variation in annotations or occlusion by fatty tissue. This explains why we found the highest intra- and interobserver variation for annotation of the azygos vein and vena cava (Dice 0.89 intraobserver variation, Dice 0.86 interobserver variation). On most frames, the vena cava and azygos vein were visible, resulting in more training data compared to the other two structures (aorta and lung), which could explain the lowest IQR found for the vena cava and azygos vein. The lung was not detected by the algorithm in two cases. Especially in the later stages of the procedure when the lung is usually not visible, the algorithm tends to generate less accurate segmentations (fifth frame in Fig. 4). This can be explained by the lack of training data with a visible lung at this specific stage. The algorithm showed the lowest accuracy and highest variation for the detection of the aorta (Dice 0.74 with an IQR of 0.86). On 7 (10%) of the frames in the test set where the aorta was present, the deep learning algorithm did not detect this structure. The aorta can be partially covered with fatty or connective tissue during RAMIE, which could have resulted in variations in annotation in these frames.

In this study, the effect of pretraining on algorithm accuracy was reported. Pretraining using the image database of ImageNet and GastroNet showed to improve algorithm accuracy. Especially for the detection of the aorta, adding pretraining resulted in a higher Dice (0.26 from scratch versus 0.74 with pretraining, *p* < 0.05). This may be explained by the lower number of training frames and because the detection task of the aorta is more complex due to visual obstruction of fatty tissue covering parts of the aorta. Large improvements due to pretraining are especially observed when the labeled dataset is limited and the segmentation task is more challenging. Furthermore, recent studies also show that the effect of pretraining on segmentation accuracy is highly task and data dependent [26]. This is in line with the results displayed in Fig. 3, which also depict that the effect of pretraining increases with a decrease in labeled data. This suggests that studies using a smaller labeled dataset and more challenging segmentation tasks could potentially benefit more from pretraining. Additionally, Fig. 3 shows that training from scratch is more unstable, showing higher variation between model performances trained on a different fraction of the training data.

High-quality annotations of the reference standard are critical to develop high-accuracy algorithms for anatomy recognition. However, the creation of large annotated datasets is time consuming, and time from expert surgeons is costly. In our experiment, a medical doctor and surgical PhD candidate in esophageal surgery showed high concordance with annotations of the expert Upper GI surgeon. This indicates that well-trained surgical PhD-researchers are able to perform reference annotation with supervision sessions and adaptation by experts. In contrast to other medical deep learning applications, ‘perfect’ accuracy approaching 100% by the deep learning model is not strictly required for the currently intended model to be of clinical added value, as the ultimate aim is to offer intraoperative surgical guidance. For example, in studies focusing on tumor detection or diagnosis, the aim is to achieve 100% accuracy [27, 28]. In our case, the ultimate goal is to apply intelligent intraoperative surgical guidance to support surgeons in their anatomy recognition and surgical orientation. Detection of some part of the structure could already provide additional guidance. Nevertheless, higher accuracy of the CNN segmentation model is likely to offer better intelligent surgical guidance to surgeons.

Although the number of studies reporting on deep learning-based anatomy recognition on surgical videos has increased over the past years, still only few studies have been published on this topic. The first report of computer-aided anatomy recognition in esophageal surgery aimed at automatic recognition of the laryngeal recurrent nerve and it reached a Dice of 0.58 [29]. As the Dice score is dependent on the size of the target structure and difficulty of the recognition task, a lower Dice score is expected for the detection of smaller structures in comparison with large and well-defined organs. Previous studies mostly focused on laparoscopic cholecystectomy with segmentation of the gallbladder and liver and reported a Dice of 0.92 for the liver and intersection of union (IoU) of 88.5% for the gallbladder [30, 31]. Both studies used approximately 200 surgical videos and 2000 frames, which were annotated by junior or expert surgeons. One study used pretraining on their algorithm [31]. Their obtained accuracy is comparable to the accuracy in the current study.

In a recent systematic review, a literature overview of 23 studies based on computer-aided anatomy recognition in 992 surgical videos was provided and factors that may contribute to a high-accuracy algorithm were identified [32]. Studies using a high number of surgical videos with reference annotation by experts tended to have better quantitative scores for anatomy recognition tasks. Some of the high-accuracy algorithms were developed using pretraining [31, 33]. Additionally, pretraining resulted in better quantitative accuracy scores compared with training solely with the surgical frames. This also applied to our study, where an equivalent accuracy was reached using pretraining and only 30% of the training data compared to training from scratch using 100% of the training data. Furthermore, recent developments in the field of pretraining, with the introduction of new self-supervised learning methods and even larger datasets, are expected to further improve network performance without the addition of extra surgical-annotated data [34–37].

Computer-aided surgical navigation has potential to improve anatomical recognition and orientation of (novice) surgeons and may reduce their learning curves. Detection of key anatomical structures might reduce the incidence of injury to vital structures. With regard to RAMIE in specific, the most challenging parts of the thoracic phase include the lymphadenectomy near vital anatomical structures. Recognition of key anatomical structures can be beneficial during these essential surgical steps. In addition to recognition of anatomical structures, algorithms can identify surgical ‘go and no-go zones’ to indicate areas of high surgical risk and can propose preferred surgical dissection planes in oncological surgery, as demonstrated in recent publications [30, 38]. Another approach to anatomy recognition is intraoperative use of preoperative imaging models [39–42]. Major challenges include tissue deformations and matching the imaging models with the intraoperative view. Video-based anatomy recognition using deep learning is less likely to be impacted by this.

This study has some limitations. The video analysis was performed retrospectively in a high-volume expert RAMIE center using a highly standardized approach for the thoracic dissection to facilitate algorithm development, but this could make the algorithm performance less robust to data from other centers: it is likely that anatomical exposure of key structures in the present cohort was of high quality with minimal visual obstruction and minimal residual fascia or fatty tissue on the target structures, which facilitates the recognition tasks and algorithm development. Hence, this algorithm performance should be validated on surgical RAMIE videos performed by novice surgeons and expert surgeons in different centers with different patient populations. Furthermore, frames were manually selected, based on the criterion either one of the three anatomical structures being visible. To avoid bias in the dataset random labeling over the entire video would be recommended. Strong points of the study are the relatively large sample size of surgical videos performed according to a standardized step-wise manner, which facilitated algorithm development [43]. Reference annotations were performed under expert supervision. Moreover, we reported on the separate effects of individual modeling steps such as pretraining, number of frames, and expert annotation on the algorithm accuracy. This could be valuable for future development of high-quality anatomy recognition algorithms.

Although the proposed CNN segmentation model showed potential for real-time segmentation of key anatomical structures, the results were not yet comparable to a surgical PhD candidate and an expert surgeon. There are multiple directions for improvement. Future work could focus on creating a CNN model that leverages the spatiotemporal relation between consecutive frames in the RAMIE videos. These algorithms use segmentations on previous frames to predict the segmentation on the next frame, which is especially useful in surgical videos recorded with stable cameras, as is the case in robot-assisted surgery. Previous studies already showed superior accuracy using a clip-level-based CNN segmentation model [44]. However, adding the spatiotemporal relation increases the complexity of the network and potentially increases the inference time, which could hinder real-time application of the algorithm. Information on surgical phase can help with the expected anatomy and may improve algorithm accuracy of anatomy recognition. Future studies require more diverse datasets to improve the generalizability of developed algorithms that are robust to various anatomical situations, preferably with videos from different patient populations and multiple surgeons using various surgical techniques or a different sequence in the surgical steps to perform RAMIE. Furthermore, qualitative assessment by expert surgeons on prospective videos is crucial to further assess clinical applicability.

In conclusion, this study shows that deep learning-based semantic segmentation has potential for anatomy recognition in thoracoscopic RAMIE video frames. The developed algorithm was able to segment video frames in real time. Deep learning-based anatomy recognition has the potential to improve surgical orientation, anatomical recognition, and surgical training for novice surgeons in future. Prospective studies are necessary to assess applicability in clinical practice.

## Supplementary Information

Below is the link to the electronic supplementary material.

Demonstration of the performance of the deep learning algorithm recognizing the vena cava or vena azygos (blue), aorta (red), and the right lung (purple) in a RAMIE video, recorded with 25 frames per second. Supplementary file1 (MP4 1160 kb)

Supplementary file2 (DOCX 13 kb)

Figure A: This section of the appendix gives a more elaborate explanation of the EfficientNet-B0 architecture. The total number of layers in EfficientNet-B0 is 237. These layers can be made from 5 different models as shown in Fig.A.1. These modules are combined to create sub-blocks as shown in Fig.A.2. Module 1 is used as a starting point for the sub-block 1, whereas module 2 is used as starting point for the other two sub-blocks. Module 3 works as a skip connection in all sub-blocks. Furthermore, Module 4 is used as a skip connection in the first sub-blocks. Eventually, module 5 connects the skip connection of the previous sub-block. Subsequently, a combination of these sub-blocks leads to the final EfficientNet-B0 architecture (Fig.A.3.). Sub-block 1 is only used as the first sub-block in the first general block. All the other blocks start with sub-block 2. Sub-block 3 is used in all other blocks except for the first one. Fig.A.3. shows the complete architecture of the EffiecentNet-B0. The red brackets visualized at the bottom of block 6 indicate that the sequencing repeats itself twice. Supplementary file3 (JPG 1900 kb)

Supplementary file4 (JPG 1478 kb)

Supplementary file5 (JPG 1722 kb)
