# Supplementary material for: Deep learning-based recognition of key anatomical structures during robot-assisted minimally invasive esophagectomy
Source: Surg Endosc. 2023 Mar 22;37(7):5164–75. doi: 10.1007/s00464-023-09990-z (PMC10322962; doi:10.1007/s00464-023-09990-z)
Supplement: Supplementary file 2 — Supplementary file2 (DOCX 13 kb) [file 464_2023_9990_MOESM2_ESM.docx]

# Appendix:

## A. Network architecture

(FIGURE A)

## B Cross-entropy loss function:

The cross-entropy loss $\mathcal{L}_{BCE}$ was defined as:

$$\mathcal{L}_{BCE}=-\frac{1}{N}\sum_{i=1}^{N} \left( y_{i}\ln\left( \hat{y}_{i} \right)+\left( 1-y_{i} \right)\ln\left( 1-\hat{y}_{i} \right) \right)$$

N represents the number of training samples, 𝑦𝑖 is the class label, $\hat{y}_{i}$ is the predicted probability for the positive class.

## C Pretraining details:

All pretrained weights of the encoder were frozen, only the weights of the decoder will be updated, as is common practice in transfer learning approaches^24,25^. Additionally, finetuning ends with unfreezing all parameters and training the network with a learning rate of 0.001, for a maximum of 1000 epochs, as is common practice for transfer learning. The learning rate was halved after 10 consecutive epochs without improvement of the validation loss. Early stopping was employed after 100 consecutive epochs without improvement of the validation loss. The weights of the epoch with the lowest validation loss were saved.
