# Supplementary figures and images for: Deep learning-based recognition of key anatomical structures during robot-assisted minimally invasive esophagectomy
Source: Surg Endosc. 2023 Mar 22;37(7):5164–75. doi: 10.1007/s00464-023-09990-z (PMC10322962; doi:10.1007/s00464-023-09990-z)

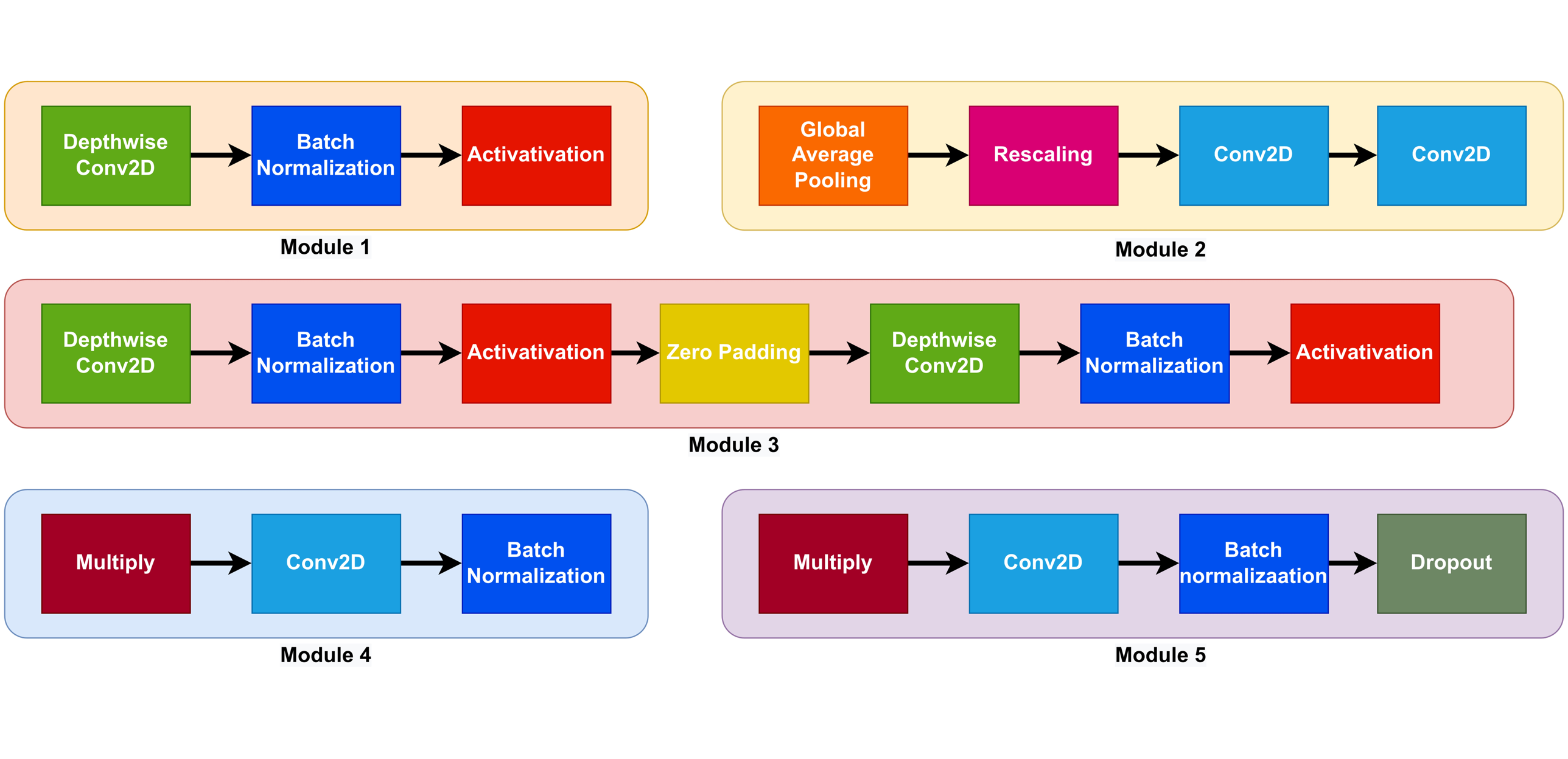

Supplement: Supplementary file 3 — Figure A: This section of the appendix gives a more elaborate explanation of the EfficientNet-B0 architecture. The total number of layers in EfficientNet-B0 is 237. These layers can be made from 5 different models as shown in Fig.A.1. These modules are combined to create sub-blocks as shown in Fig.A.2. Module 1 is used as a starting point for the sub-block 1, whereas module 2 is used as starting point for the other two sub-blocks. Module 3 works as a skip connection in all sub-blocks. Furthermore, Module 4 is used as a skip connection in the first sub-blocks. Eventually, module 5 connects the skip connection of the previous sub-block. Subsequently, a combination of these sub-blocks leads to the final EfficientNet-B0 architecture (Fig.A.3.). Sub-block 1 is only used as the first sub-block in the first general block. All the other blocks start with sub-block 2. Sub-block 3 is used in all other blocks except for the first one. Fig.A.3. shows the complete architecture of the EffiecentNet-B0. The red brackets visualized at the bottom of block 6 indicate that the sequencing repeats itself twice. Supplementary file3 (JPG 1900 kb) [file 464_2023_9990_MOESM3_ESM.jpg]

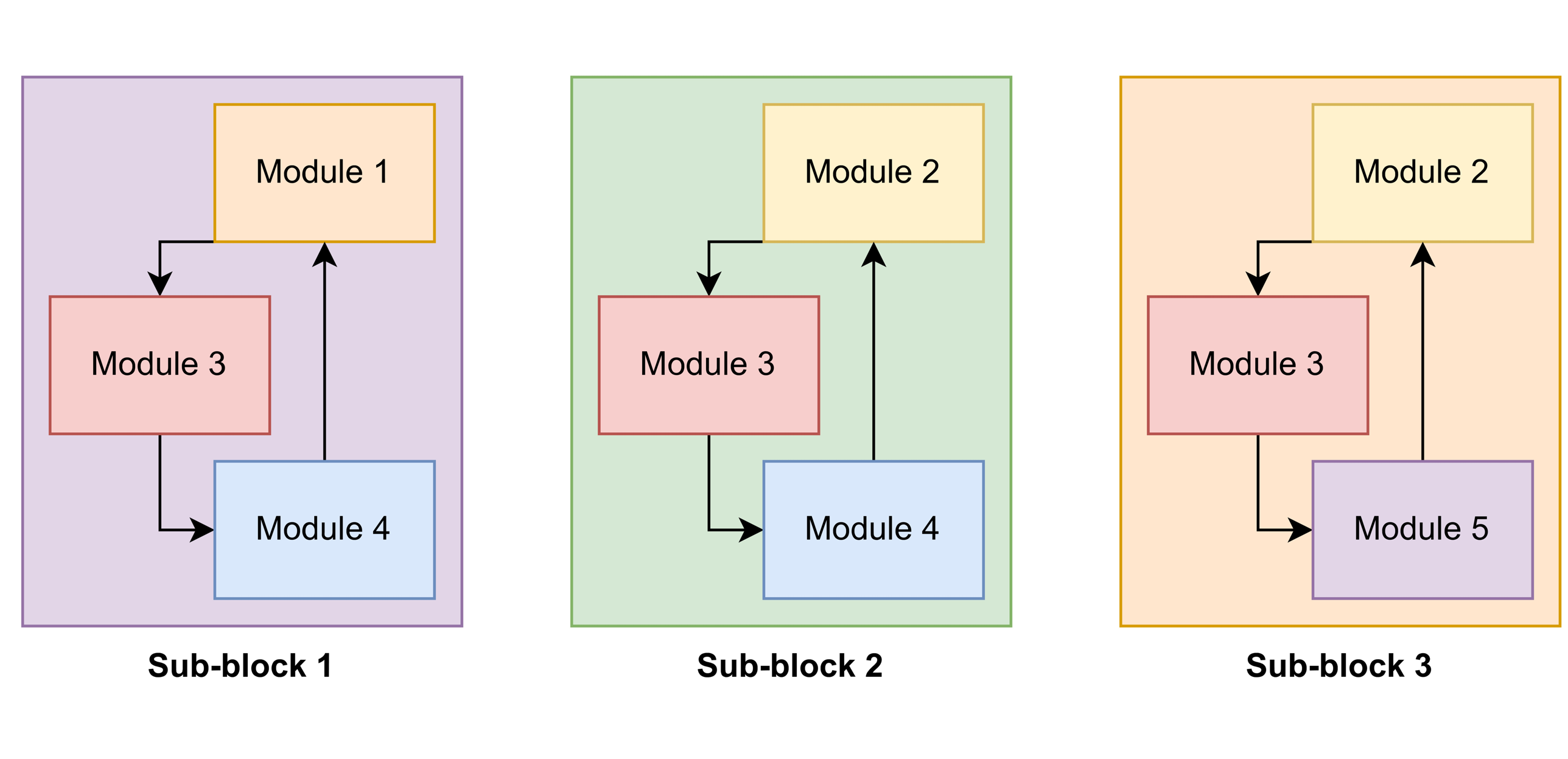

Supplement: Supplementary file 4 — Supplementary file4 (JPG 1478 kb) [file 464_2023_9990_MOESM4_ESM.jpg]

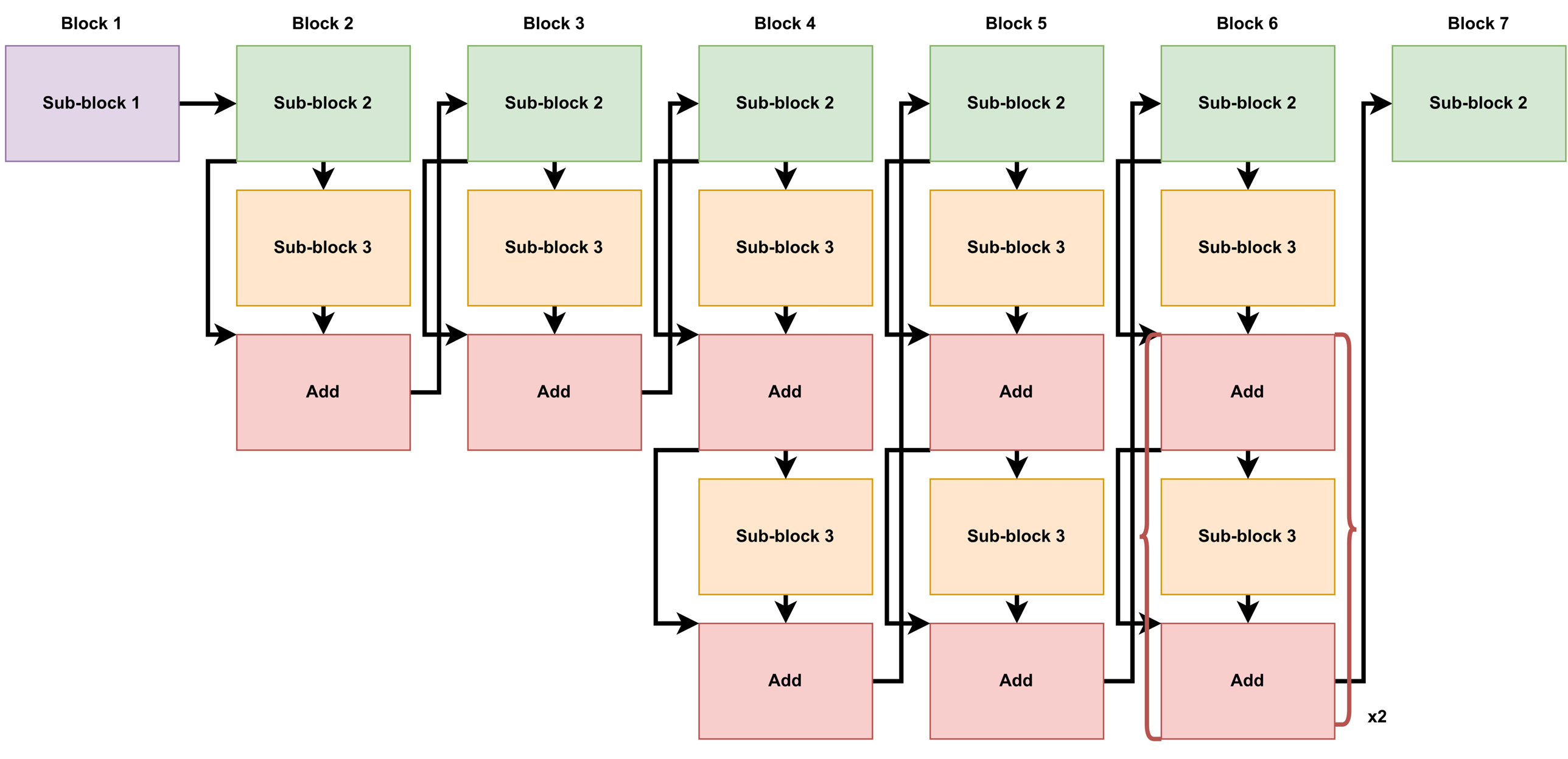

Supplement: Supplementary file 5 — Supplementary file5 (JPG 1722 kb) [file 464_2023_9990_MOESM5_ESM.jpg]
